# Co-occurrence and genetic contexts of NDM-1, KPC-2, IMP-1, and AFM-1 carbapenemase genes in a critically ill patient

**DOI:** 10.3389/fcimb.2026.1973471

**Published:** 2026-10-16

**Authors:** Yan Zhang, Zhirong Zhang, Senhong Ying, Sanrong Wang, Rong Xiang

**Affiliations:** 1 Precision Medicine Center, The Second Affiliated Hospital of Chongqing Medical University, Chongqing, China; 2 Department of Critical Care Medicine, The Second Affiliated Hospital of Chongqing Medical University, Chongqing, China; 3 Gut Microbiome Diagnosis and Treatment Center, The Second Affiliated Hospital of Chongqing Medical University, Chongqing, China; 4 Chongqing Municipality Clinical Research Center for Geriatrics and Gerontology, Chongqing, China; 5 Department of Rehabilitation Medicine, The Second Affiliated Hospital of Chongqing Medical University, Chongqing, China; 6 Western (Chongqing) Institute for Digital-Intelligent Medicine, Chongqing National Biomedicine Industry Park, Chongqing, China

**Keywords:** *bla*_AFM-1_, *bla*_NDM-1_, *bla*_IMP-1_, *bla*_KPC-2_, carbapenemases, global antimicrobial resistance

## Abstract

**Background:**

Carbapenem-resistant organisms are increasingly reported in clinical settings, yet the simultaneous detection of multiple carbapenemase genes within a single specimen remains rare.

**Methods:**

Metagenomic next-generation sequencing (mNGS) and whole-genome sequencing (WGS) were performed on a bronchoalveolar lavage fluid (BALF) sample from a patient with severe pneumonia.

**Results:**

Four carbapenemase genes—*bla*_AFM-1_, *bla*_NDM-1_, *bla*_IMP-1_, and *bla*_KPC-2_—were simultaneously detected in a single specimen. Three carbapenem-resistant isolates were recovered: an ST11 *K. pneumoniae* carrying *bla*_KPC-2_ and virulence-associated genes, an *S. algae* strain with an assembly-based tandem chromosomal *bla*_NDM-1_ arrangement, and a *P. stutzeri* strain co-harboring *bla*_AFM-1_ and *bla*_IMP-1_. Genomic analysis revealed complex mobile genetic contexts associated with these resistance determinants.

**Conclusion:**

The coexistence of four carbapenemase genes within one clinical sample highlights the complexity of polymicrobial carbapenem resistance and underscores the importance of genomic surveillance.

## Introduction

Antimicrobial resistance (AMR) is a major global health challenge, exacerbated by the emergence and dissemination of carbapenem-resistant organisms (CROs) (Tacconelli et al., 2018; Antimicrobial Resistance C, 2022). Carbapenems, once considered the most reliable last line of defense against severe Gram-negative bacterial infections, are increasingly compromised by resistant pathogens (Tzouvelekis et al., 2012). The growing prevalence of carbapenemase-producing organisms, particularly among Enterobacteriaceae and non-fermenting Gram-negative bacilli, has severely restricted treatment options worldwide and is associated with alarming mortality rates exceeding 50% in some regions (Logan and Weinstein, 2017).

Carbapenem resistance is primarily mediated by carbapenemase enzymes encoded by genes such as *bla*_KPC_, *bla*_NDM_, *bla*_IMP_, *bla*_VIM_, and *bla*_OXA-48_ (Meletis, 2016; Tamma et al., 2017). These resistance determinants are frequently located on mobile genetic elements like plasmids, transposons, and integrons, facilitating cross-border horizontal gene transfer (HGT) among diverse bacterial species (Rozwandowicz et al., 2018; Wu et al., 2019). The co-occurrence of multiple carbapenemase genes within individual bacterial isolates represents an escalating global concern, as it potentially leads to higher resistance levels and undermines combination antibiotic therapies across healthcare systems worldwide (Gao et al., 2020).

Metagenomic next-generation sequencing (mNGS) has transformed our ability to detect pathogens and characterize antimicrobial resistance profiles directly from clinical samples across international boundaries (Forbes et al., 2017; Miller and Chiu, 2021). This culture-independent approach enables identification of pathogens and resistance genes in polymicrobial infections, which often present complex diagnostic challenges using traditional methods (Miller et al., 2013; Charalampous et al., 2019). Despite these technological advances, the simultaneous presence and genetic contexts of multiple carbapenemase genes within single clinical specimens remain underexplored, particularly in polymicrobial infections involving both Enterobacteriaceae and non-fermenting Gram-negative bacilli (Bes et al., 2021; Rodrigues et al., 2024).

Most international studies have focused on single carbapenemase-producing organisms or the prevalence of individual carbapenemase genes in clinical isolates from specific geographical regions (van Duin and Doi, 2017; Jin et al., 2022). Simultaneous detection of multiple carbapenemase genes within a single clinical specimen remains rare. Their coexistence may enhance bacterial survival under antibiotic pressure, promote the emergence of highly resistant strains through genetic recombination, and complicate infection control and treatment (Potron et al., 2015; Logan and Weinstein, 2017).

In this study, we report a clinical case involving the simultaneous detection of four distinct carbapenemase genes—*bla*_AFM-1_, *bla*_NDM-1_, *bla*_IMP-1_, and *bla*_KPC-2_—in a single bronchoalveolar lavage fluid (BALF) sample from a patient with severe pneumonia and acute respiratory distress syndrome (ARDS). We employed mNGS and whole-genome sequencing (WGS) to comprehensively characterize the pathogens present and elucidate the genetic contexts and potential mechanisms underlying the dissemination of these antimicrobial resistance genes (ARGs).

It provides insights into the genetic contexts and potential mobility of resistance genes detected in the same clinical specimen (Kong et al., 2017; Zhang et al., 2017). It also illuminates the complexities involved in treating polymicrobial infections where pathogens may possess overlapping and synergistic resistance mechanisms, complicating antimicrobial therapy worldwide (Tamma et al., 2012). Additionally, it emphasizes the importance of implementing comprehensive global genomic surveillance networks to detect and monitor the spread of highly resistant organisms, informing international infection control measures and guiding effective therapeutic strategies (Chen et al., 2017).

Our findings contribute to a deeper understanding of the molecular epidemiology of carbapenem resistance as a transnational health challenge and the complex obstacles posed by MDR bacterial infections in clinical practice worldwide. By elucidating the genetic architectures of multiple carbapenemase genes within the same clinical specimen, this study underscores the urgent need for coordinated international research and development of novel antimicrobial agents and therapeutic approaches to combat the growing global threat of antibiotic resistance.

## Materials and methods

### Sample collection and processing

A BALF sample was collected from a male patient admitted to the intensive care unit of a tertiary hospital in Chongqing, China, with aspiration pneumonia and ARDS. Clinical diagnoses also included sepsis and infection-associated multiorgan dysfunction. *K. pneumoniae*, *P. stutzeri* and *S. algae* were considered clinically significant based on the clinical assessment. The patient received intravenous imipenem/cilastatin and vancomycin throughout hospitalization and subsequently died. Following standardized international protocols, a total of 100 mL sterile saline was instilled and aspirated via bronchoscopy. The BALF sample was divided into two aliquots: one preserved at –80 °C for mNGS analysis and one processed for bacterial culture and identification (**Supplementary Table 1**). The study was approved by the Ethics Committee of the Second Affiliated Hospital of Chongqing Medical University, and written informed consent was obtained from the patient.

### Metagenomic sequencing and analysis

- Sample Processing and DNA Extraction: BALF samples were processed within 24 hours of collection following international best practices. Up to 1.5 mL of sample was transferred to sterile tubes and agitated with glass beads to disrupt microbial cell walls. DNA extraction was performed using a magnetic bead-based nucleic acid extraction kit (Genskey, China) according to the manufacturer’s instructions. DNA quality and quantity were assessed using a Qubit 4.0 fluorometer (Invitrogen, USA), adhering to global laboratory standards.
- Library Construction: Libraries were prepared using a standardized library preparation kit (Genskey, China) involving DNA fragmentation, end repair, adapter ligation, PCR amplification, and purification. Libraries with concentrations above 1 ng/µL and fragment sizes between 240–350 bp were pooled and sequenced on the MGI200 platform using DNA Nanoball technology, generating 10,097,330 single-end 50-bp raw reads for this library.
- Data Analysis: Raw reads were processed using Trimmomatic to remove low-quality sequences. Clean reads were aligned to human reference genomes (hg19 and hg38) using BWA-MEM, and non-human reads were retained for microbial analysis. These reads were aligned to a reference database containing genomes of viruses, bacteria, fungi, and parasites. Microbial identification required at least three non-overlapping reads for bacteria (excluding *Mycobacterium*, *Nocardia*, and *Legionella pneumophila*) that were either undetected in negative controls or present at counts at least 10 times higher than in controls. Reads were aligned to NCBI AMRFinderPlus nucleotide references using BWA-backtrack. Full-length 50-bp alignments with no more than one mismatch were retained after quality filtering. Read counts, coverage breadth and mean depth were calculated for *bla*_KPC-2_, *bla*_NDM-1_, *bla*_AFM-1_ and *bla*_IMP-1_. These results provide specimen-level gene support. Gene coordinates were identified by full-length matching to the deposited assemblies.

### Bacterial isolation, identification, and whole-genome sequencing

To isolate carbapenem-resistant bacteria, the BALF sample was diluted and incubated in Luria-Bertani broth overnight at 37 °C. Cultures were streaked onto LB agar plates containing 0.5 µg/mL of imipenem for selective culture. Antimicrobial susceptibility of the three isolates was assessed using standard minimum inhibitory concentration (MIC) testing. Selected colonies underwent PCR screening for carbapenemase genes (*bla*_NDM-1_, *bla*_KPC-2_, *bla*_IMP-1_, and *bla*_AFM-1_) using internationally validated primers (Monteiro et al., 2012; Qin et al., 2023). DNA was extracted from strains harboring the carbapenemase genes, and WGS was performed using Oxford Nanopore Technologies (ONT) on the MinION platform according to global best practices for genomic analysis. Basecalling was performed with Guppy (Wick et al., 2019), and genome assemblies were generated using Flye (Kolmogorov et al., 2019). Assemblies were polished with Medaka (https://github.com/nanoporetech/medaka) to enhance accuracy.

### Bioinformatics analysis workflow

The assembled bacterial genomes were annotated using Prokka v1.13 (--kingdom Bacteria --cpus 20) (Seemann, 2014). ARGs in assembled sequences were screened using ABRicate v1.0.1 with the NCBI and ResFinder databases (Florensa et al., 2022), using the default thresholds of ≥80% nucleotide identity and ≥80% reference-gene coverage (--minid 80 --mincov 80). Insertion sequences were detected using ISfinder (Siguier et al., 2006). PlasmidFinder and VFDB screening was repeated with the same ABRicate thresholds. The local NCBI, ResFinder, PlasmidFinder and VFDB builds were dated 4 November 2023 and contained 5,386, 3,077, 460 and 2,597 sequences, respectively. *K. pneumoniae* sequence type, virulence genotype and K/O loci were reassessed using Kleborate v3.2.4 (--preset kpsc --trim_headers), with Kaptive v3.1.0 in the analysis environment. The isolates were taxonomically classified at the species level using GTDB-Tk v2.4.0, release r220 (classify_wf --extension fna --cpus 32 --skip_ani_screen) (Chaumeil et al., 2019). To elucidate the genomic context and arrangement of carbapenem resistance genes, neighboring genes and flanking sequences were analyzed using Geneious Prime software. Graphical representations of bacterial genome structures were generated using Easyfig (Sullivan et al., 2011). Assembly statistics were obtained using QUAST v5.0.2 (--threads 8 --min-contig 500 --no-plots). Ungapped contig counts and contig N50 were calculated after splitting deposited sequences at N runs; nominal depth was the reported yield of approximately 1 Gb divided by total assembly length, rather than read-mapping coverage.

## Results

### Complex antibiotic resistance patterns revealed by metagenomic analysis

Metagenomic analysis of the patient’s BALF detected *Pseudomonas stutzeri*, *Klebsiella pneumoniae*, *Shewanella algae*, and *Elizabethkingia anophelis*. The sample contained a diverse array of ARGs spanning multiple categories, including beta-lactams *bla*_AFM-1_, *bla*_CTX-M-65_, *bla*_NDM-1_, *bla*_SHV-66_, *bla*_OXA-55_, *bla*_IMP-1_, *bla*_KPC-2_, *bla*_OXA-246_; aminoglycosides (*aph(3’)-Ia*, *aph(3’)-Ib*, *aph(3’)-IIIa*, *aph(3’)-Via*, *aph(3’’)-Ib*, *aph (*6)*-Id*, *ant(2’’)-Ia*, *aac(6’)-Ib*, *aac(6’)-Il*, *aac(6’)-IIa*); sulfamethoxazole (*sul1*, *sul2*); trimethoprim (*dfrA27*, *dfrA6*, *dfrB3*); tetracyclines (*tet(C)*, *tet(G)*, *tet(A)*); chloramphenicol (*floR*, *cmlA8*); rifampicin (*arr-3*); fluoroquinolones (*qnrVC1*); and macrolides (*mphA*).

Furthermore, various virulence-associated genes were detected, including those involved in iron acquisition (*iucA*, *iroN*, *ybtX*, *ybtP*, *ybtU*, *ybtT*, *ybtE*), fimbriae formation (*rmpA*, *rmpA2*, *fimB*, *fimE*, *fimA*, *fimI*, *fimC*, *fimD*, *fimF*, *fimG*, *fimH*, *fimK*, *mrkF*, *mrkD*, *mrkC*, *mrkB*, *mrkA*, *mrkH*, *mrkI*, *mrkJ*), and outer membrane protein synthesis (*exeE*, *tapW*). The presence of these genes suggests a virulence-associated genetic profile.

Using selective culturing techniques, three carbapenemase-producing strains were isolated: *K. pneumoniae* strain CQPMC-KpYD carrying *bla*_KPC-2_, *S. algae* strain CQPMC-SheW1 with *bla*_NDM-1_, and *P. stutzeri* strain CQPMC-PStu harboring both *bla*_IMP-1_ and *bla*_AFM-1_. Targeted BALF reanalysis yielded 148, 477, 232 and 325 reads compatible with *bla*_KPC-2_, *bla*_NDM-1_, *bla*_AFM-1_ and *bla*_IMP-1_, respectively; corresponding coverage breadths were 99.43%, 99.75%, 99.88% and 99.73%, and mean depths were 8.39×, 29.34×, 14.43× and 21.93×. Species-level signals exceeded the same-chip DNA/PCR and host-depleted DNA/PCR negative controls. All five deposited carbapenemase loci matched their full reference sequences exactly.

The MICs (µg/mL) for imipenem, meropenem and ertapenem were ≥16 in all three isolates. Corresponding MIC values were ≥128 for piperacillin/tazobactam, ≥64 for ceftazidime, cefepime, amikacin and trimethoprim-sulfamethoxazole, ≥16 for gentamicin, and ≥4 for ciprofloxacin.

Yield-based nominal sequencing depths were 169×, 204× and 191× for KpYD, SheW1 and PStu, respectively, with read N50 values of 16,436, 17,374 and 16,338 bp. Their assemblies comprised 4, 1 and 3 ungapped contigs, with total lengths of 5,930,020, 4,891,834 and 5,242,803 bp and contig N50 values of 5,584,340, 4,891,834 and 5,236,254 bp, respectively.

### Genomic insights into ST11 *K. pneumoniae* carrying virulence-associated genes

Comprehensive genomic analysis identified strain CQPMC-KpYD as an ST11 strain carrying virulence-associated genes, a lineage known for its international dissemination and contribution to the global AMR crisis. WGS revealed that the *bla*_KPC-2_ gene (CP170657.1:122,231–123,112 [+]), along with other resistance genes such as *bla*_CTX-M-65_, *bla*_TEM-1_, *rmtB*, and *bla*_SHV-12_, is located on a 132,742 bp plasmid designated pCQPMC-KpYD1. This plasmid harbors 172 predicted open reading frames (ORFs) with an average GC content of 53.4% and contains IncFII(pHN7A8) and IncR replicons in the repeat PlasmidFinder screen.

BLAST database comparisons showed that pCQPMC-KpYD1 shares 100% coverage and 99.99% identity with the *K. pneumoniae* plasmid pCRKP-35_KPC (GenBank accession number CP102639), isolated from a patient’s blood sample in China in 2019. Further searches identified 45 plasmids with over 99% identity and coverage to pCQPMC-KpYD1, all from *K. pneumoniae* isolates across various regions in China—43 from hospital patients and 2 from animals. These similarities suggest that related plasmid backbones may contribute to *bla*_KPC-2_ dissemination.

The CQPMC-KpYD isolate also harbors virulence genes including *iucABCD*, *iutA*, *rmpA*, *rmpD*, *rmpC*, located on a 200,970 bp IncHI1B-IncFIB hybrid plasmid lacking additional antibiotic resistance genes. Genetic analysis revealed that the core structure of the *bla*_KPC-2_ gene region, termed variable region 1 (VR1), comprises IS*26*-ΔTn3-IS*Kpn27*-*bla*_KPC-2_-IS*Kpn6*-*korC*-*orf*-*klcA*-Δ*repB*. VR1 closely resembles the *bla*_KPC-2_ core structure on plasmid pJNKPN52_kpc_fosA (MZ709016) from *K. pneumoniae* strain JNKPN52, differing primarily in the upstream IS*26* element (**Figure 1**). Capsule and O-locus predictions were KL64 (predicted K64; 99.93% identity) and OL2α.1 (predicted O2α; 99.03% identity), both Typeable. Kleborate reported a partial *rmpA2* call (*rmpA2_3-47%*).

**Figure 1:**
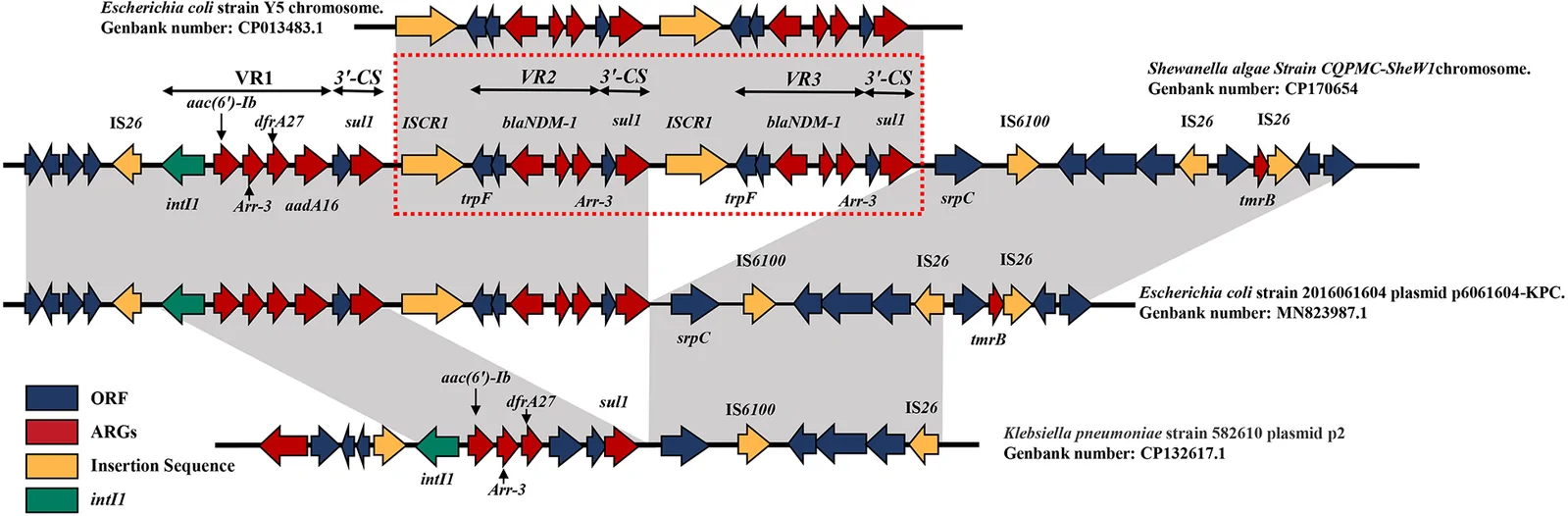
Comparative genomic analysis of the genetic context of *bla*_KPC-2_ in plasmid pCQPMC-KpYD1 and other plasmids. Genes are depicted as arrows, color-coded according to their functional classification. Shaded regions indicate syntenic regions with >95% nucleotide identity. Antibiotic resistance genes are highlighted in red, insertion sequences in yellow, mercury resistance operons in pink, and other functional genes in blue.

Two additional resistance gene clusters adjacent to *bla*_KPC-2_, designated VR2 and VR3, were identified. VR2 is a mercury resistance (*mer*) gene cluster organized as *merR-merT-merP-merC*-*merA-merD-merE*, flanked by IS*5057* and *TnpA*. VR3 contains the *bla*_SHV-12_ gene, enclosed by *TnAs1* and IS*26*. BLASTn analysis indicated that VR1 and VR2 share over 99% similarity with the *E. coli* plasmid pHKN-KPC (MW191860), while VR3 shows similarity with plasmid pKP1650-2 (CP109950) from *K. pneumoniae* strain KP1650 (**Figure 1**). These genetic contexts suggest potential roles for transposition and recombination in the genetic diversity and mobility of *bla*_KPC-2_-carrying plasmids.

### *Shewanella algae* strain CQPMC-SheW1 harboring putative tandem ISCR1-NDM-1 elements

Comprehensive genomic analysis of *S. algae* strain CQPMC-SheW1, which harbors the *bla*_NDM-1_ gene associated with international outbreaks of carbapenem resistance, reveals a genome comprising 4,891,834 bp with a GC content of 53%. Beyond *bla*_NDM-1_, CQPMC-SheW1 carries an array of ARGs including *aac(6’)-Ib*, *arr-3*, *aac (*Tzouvelekis et al., 2012)*-IId*, *aadA16*, *aadA2*, *aph(3’)-Ia*, *bla*_OXA-55_, *dfrA12*, *dfrA27*, *floR*, *mph(A)*, *mph(E)*, *msr(E)*, *qnrA7*, *sul1*, *and tet(A)*. Additionally, the strain contains heavy metal resistance genes such as *merA*, *merC*, *merD*, *merE*, *merP*, *merR1*, and *merT*.

Using ISfinder, multiple insertion sequences were identified in CQPMC-SheW1, including IS*6100*, IS*Ec68*, IS*Prre2*, IS*Shes12*, IS*Slo2*, IS*Sod21*, IS*Spu20*, IS*Va2*, IS*Val1*, and IS*Vch1*. Analysis revealed that *bla*_NDM-1_ is located within an approximately 31 kb MDR region flanked by IS*26* elements. Within this region, the deposited chromosome assembly contained two *bla*_NDM-1_ copies in the same orientation, at CP170654.1:22,701–23,513 and 29,100–29,912 (both on the negative strand), with start positions separated by 6,399 bp in repeated ISCR1-associated contexts. The related context was ISCR1-partial *trpF*-*ble*_MBL_-*bla*_NDM-1_-ΔISAba125-*catB3*-*arr-3*-*qacEΔ1*-*sul1*.

Remarkably, a similar IS*CR1*-NDM-1 structure is observed in *E. coli* strain Y5 (CP013483.1), which shares 100% homology with the chromosomal IS*CR1*-NDM-1 element in CQPMC-SheW1. Downstream of *bla*_NDM-1_, an *IntI1* integron with a cassette of six resistance genes (*aac(6’)-Ib-arr-3-dfrA27-aadA16-qacEΔ1-sul1*) was identified. Comparative analysis indicates that the *bla*_NDM-1_ gene and its surrounding resistance cluster in CQPMC-SheW1 share up to 99% homology with plasmid p6061604-KPC (MN823987.1) from *E. coli* strain 2016061604, highlighting potential cross-species transmission (**Figure 2**).

**Figure 2:**
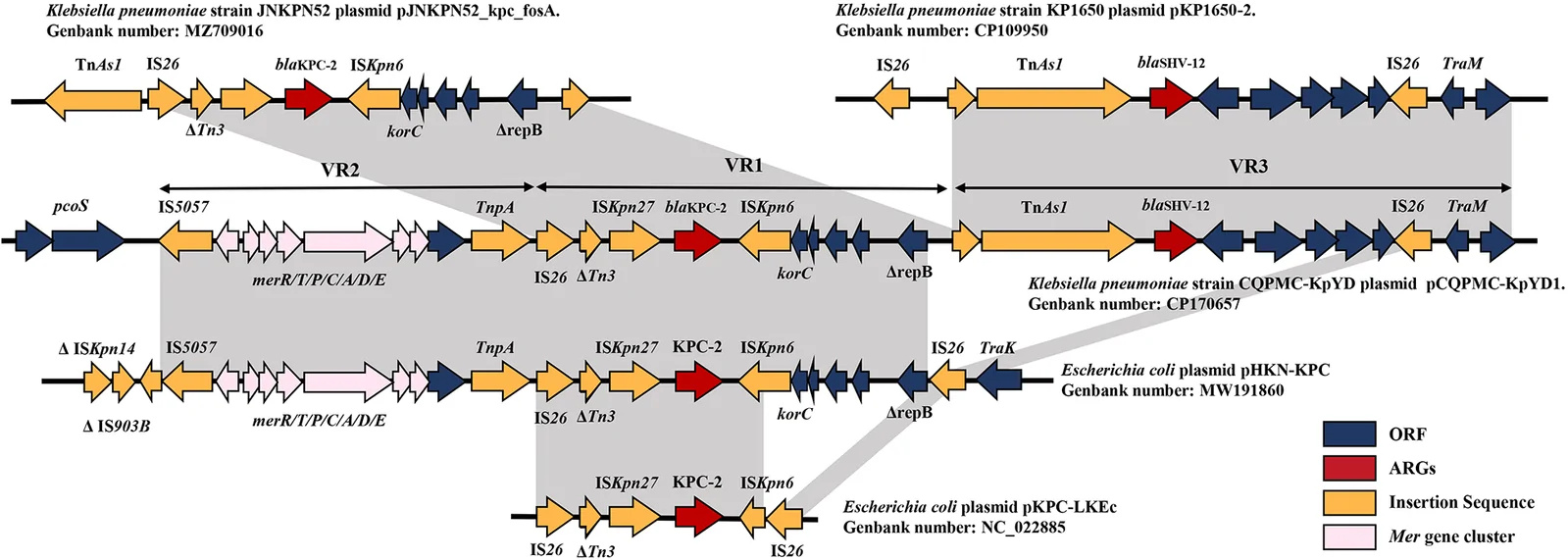
Comparative analysis of the *bla*_NDM-1_ genetic context in strain CQPMC-SheW1 and other genomes. Genes are illustrated as arrows and color-coded by functional category. Shaded regions represent homologous areas with >95% nucleotide similarity. Red arrows indicate antibiotic resistance genes, yellow represents insertion sequences, green denotes the *intI1* gene, and blue indicates other functional genes.

While plasmid p6061604-KPC contains only one complete IS*CR1*-NDM-1 element, CQPMC-SheW1 exhibits a more complex composition with two same-orientation *bla*_NDM-1_ copies, possibly resulting from recombination or transposition events. Moreover, plasmid p2 (CP132617.1) from *K. pneumoniae* strain 582610 shows high homology with the resistance cluster in CQPMC-SheW1, despite lacking the IS*CR1*-NDM-1 element. These findings underscore the complexity and diversity of ARG-associated genetic contexts among bacterial populations.

### *Pseudomonas stutzeri* strain CQPMC-PStu co-harboring *bla*_AFM-1_ and *bla*_IMP-1_

Whole-genome analysis revealed that *bla*_AFM-1_ and *bla*_IMP-1_ genes are located in the *P. stutzeri* strain CQPMC-PStu, characterized by a genome size of 5,242,803 bp and a GC content of 62.2%, encompassing 4,808 coding regions (**Figure 3C**). Further investigation identified a diverse array of additional ARGs, including *aac(6’)-IIa*, *aac(6’)-Il*, *aadA1*, *aadA25*, *aph(3’)-Ia*, *aph(3’’)-Ib*, *aph(3’)-VIa*, *aph (*6)*-Id*, *arr-3, bla*_OXA-246_, *cmlA1*, *cmlA8*, *dfrA27*, *dfrA6*, *floR*, *sul1*, and *tet(G)*. Significantly, *bla*_AFM-1_ (CP171270.1:4,408,942–4,409,745 [−]) is located within an approximately 12 kb MDR region, primarily composed of an IS*CR29* element (IS*CR29*-Δ*groEL*-*bla*_AFM-1_-*ble*_MBL_-Δ*trpF*) and two class 1 integrons (*IntI1-aac(6’)-Il-cmlA1-ant(3’’)-Ia-qacEΔ1-sul1* and *IntI1-aac(6’)-IIa-cmlA8-bla*_OXA-246_*-dfrA27-qacEΔ1-sul1*).

**Figure 3:**
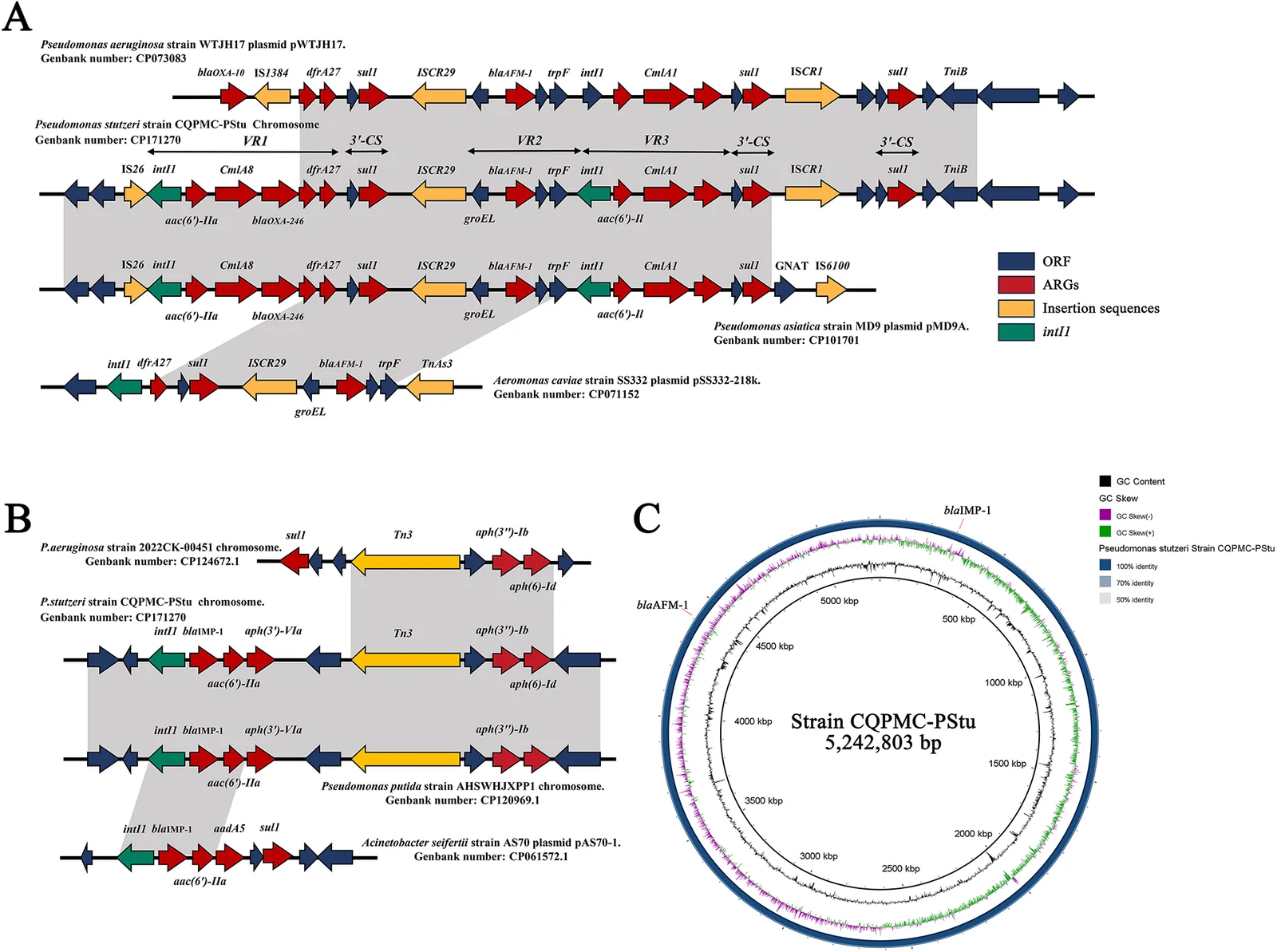
Comparative analysis of the genetic contexts of *bla*_AFM-1_ and *bla*_IMP-1_ in strain CQPMC-PStu and other genomes. **(A, B)** Genetic context analysis of *bla*_AFM-1_ and *bla*_IMP-1_ in strain CQPMC-PStu, respectively. The gray shaded areas represent BLAST homology with >95% nucleotide identity. Genes are depicted as arrows, color-coded by functional category: red indicates antibiotic resistance genes, yellow represents insertion sequences, green denotes the *intI1* gene, and blue indicates other functional genes. **(C)** Circular genomic map of strain CQPMC-PStu, generated using the BRIG tool, indicating the positions of *bla*_AFM-1_ and *bla*_IMP-1_. The innermost ring represents the GC content, while the intermediate ring displays the GC skew.

Comparative genomic analysis revealed that this MDR region is highly similar to the chromosome of clinically isolated *Pseudomonas qingdaonensis* strain L1712hy (CP129981), exhibiting 99.9% nucleotide identity. A related genetic architecture was identified in plasmid pWTJH17 (CP073083.1) of *Pseudomonas aeruginosa* strain WTJH17, where *bla*_AFM-1_ is replaced by *bla*_AFM-3_, accompanied by *bla*_OXA-10_ and IS*1384* upstream of *arr-3*. Plasmid pMD9A (CP101701) from *Pseudomonas asiatica* strain MD9 presents a similar structure; however, its variable region features GNAT and IS*6100* instead of IS*CR1*. In contrast, plasmid pSS332-218k (CP071152) from *Aeromonas caviae* strain SS332 demonstrates significant variations, notably lacking *aac(6’)-IIa*, *cmlA8*, and *bla*_OXA-246_ downstream of IS*CR29* and substituting *dfrA27* with *dfrA5*, resulting in a distinct class 1 inte*gron* (**Figure 3A**).

In addition to the *bla*_AFM-1_ gene, the *P. stutzeri* strain CQPMC-PStu also harbors the *bla*_IMP-1_ gene. Analysis revealed that *bla*_IMP-1_ (CP171270.1:303,088–303,828 [+]) is located within a truncated class 1 integron arranged as *IntI1*-*bla*_IMP-1_-*aac(6’)-IIa*-*aph(3’)-VIa* (**Figure 3B**). This structure closely resembles integron In86 (AJ640197), with the key difference being the replacement of the *aacA1* gene by *aph(3’)-VIa* in CQPMC-PStu. Adjacent to this integron is a Tn*5393c*-like transposon (*Tn3*-*orf-aph(3’’)-Ib*-*aph* (Tamma et al., 2017)*-Id*).

Notably, this MDR region exhibits high homology with the chromosome of *P. putida* strain AHSWHJXPP1 (CP120969.1), demonstrating 100% coverage and 99% identity, suggesting potential ARG dissemination within the *Pseudomonas* genus. Additionally, *P. putida* strain AHSWHJXPP1 also carries both *bla*_AFM-1_ and *bla*_IMP-1_. A similar genomic composition, including *bla*_AFM-1_ and *bla*_IMP-45_, has been discovered in plasmids of three *P. aeruginosa* strains (CP129201, CP104871, and CP061377). These findings reveal related resistance-associated regions across several bacterial species, suggesting potential mobility of the associated determinants.

## Discussion

This study reports simultaneous detection of four distinct carbapenemase genes (*bla*_AFM-1_, *bla*_NDM-1_, *bla*_IMP-1_, and *bla*_KPC-2_) in a single clinical sample. The mobile-element-associated genetic contexts of these co-occurring genes suggest their potential mobility.

### Virulence-associated genotype of ST11 *K. pneumoniae*

The identification of *K. pneumoniae* strain CQPMC-KpYD as an ST11 strain carrying virulence-associated genes is particularly concerning from a global health perspective. ST11 is a predominant clone associated with carbapenem resistance in *K. pneumoniae* and is linked to hospital outbreaks and high mortality rates across multiple continents. The presence of virulence genes such as *iucABCD*, *iutA*, *rmpA*, *rmpD* and *rmpC*, together with a partial *rmpA2* call, indicates a virulence-associated genotype. These genes have been associated with increased virulence, siderophore production, and hypermucoviscosity in previous studies, but their detection here does not establish a hypervirulent phenotype (Lam et al., 2018; Russo and Marr, 2019).

The IS-associated genetic context of the *bla*_KPC-2_ plasmid is consistent with potential mobility (Zhou et al., 2024). The high similarity of this plasmid to others indicates related genetic contexts, warranting continued surveillance (Partridge et al., 2018).

### Role of *Shewanella algae* in resistance gene dissemination

The deposited *S. algae* CQPMC-SheW1 chromosome assembly contained tandem copies of *bla*_NDM-1_, a finding consistent with the role of environmental bacteria as reservoirs for ARGs (Wu et al., 2019). *Shewanella* species are increasingly recognized for their capacity to acquire and disseminate resistance genes (Li et al., 2022; Wen et al., 2022). The two *bla*_NDM-1_ copies indicate a repeated resistance-gene arrangement in the deposited sequence (Toleman et al., 2006). IS*CR1* elements are known to mobilize adjacent genes through rolling-circle transposition, facilitating the horizontal spread of resistance (Partridge, 2011). The genetic similarity of the MDR region to plasmids found in *E. coli* and *K. pneumoniae* suggests potential interspecies mobility of ARGs (Mari-Almirall et al., 2021).

### Co-occurrence of *bla*_AFM-1_ and *bla*_IMP-1_ in *Pseudomonas stutzeri*

The *P. stutzeri* strain CQPMC-PStu co-harbored *bla*_AFM-1_ and *bla*_IMP-1_, genes encoding metallo-β-lactamases that confer high-level resistance to carbapenems (Zhang et al., 2021; Zhang et al., 2023). The location of *bla*_AFM-1_ within an MDR region composed of IS*CR29* and class 1 integrons suggests potential mobility (Martinez et al., 2015). Similarly, the arrangement of *bla*_IMP-1_ within a truncated class 1 integron mirrors structures found in other clinical isolates, suggesting a conserved mechanism of resistance gene acquisition (Umeda et al., 2021). The high homology to other *Pseudomonas* species suggests a shared resistance framework and potential intra-genus ARG exchange (Fan et al., 2016).

### Clinical and global public health implications

Co-detection of multiple carbapenemase genes in one specimen warrants careful clinical and microbiological interpretation (Tacconelli et al., 2018). The associated mobile genetic elements suggest potential mechanisms of resistance-gene mobility (Mendelson and Matsoso, 2015). These findings highlight the value of genomic surveillance for characterizing complex resistance-gene contexts (Laxminarayan et al., 2016). Such surveillance can inform infection-control and antimicrobial-stewardship efforts (Bonomo et al., 2018).

This single-case study describes the co-detection and genetic contexts of four carbapenemase genes. The absence of direct transfer experiments and longitudinal evidence limits inference of HGT within the patient. The contribution of each organism and its resistance determinants to the fatal outcome could not be established in this single patient with severe illness and multiorgan dysfunction. BALF short reads do not establish isolate origin or copy-specific coverage; independent long-read validation across the SheW1 repeat junctions was not established, and species-level controls do not specifically exclude index/carryover artifacts.

## Conclusions

The simultaneous detection of four carbapenemase genes in a single clinical specimen illustrates the complexity of polymicrobial carbapenem resistance. Comprehensive genomic analysis provides important insights into resistance gene organization and potential horizontal transfer. Strengthened surveillance and infection control strategies are required to mitigate the spread of carbapenem-resistant pathogens. Understanding the genetic contexts and mechanisms of ARG dissemination is essential for guiding clinical treatment and mitigating the spread of carbapenem-resistant pathogens.

## Data Availability

The datasets presented in this study can be found in online repositories. The names of the repository/repositories and accession number(s) can be found below: https://www.ncbi.nlm.nih.gov/, PRJNA1164473 https://www.ncbi.nlm.nih.gov/, PRJNA1164442.

## Supplementary material

Supplementary Table 1 Clinical information of the patient.
